# Quantitative ultrasound assessment of breast tumor response to chemotherapy using a multi-parameter approach

**DOI:** 10.18632/oncotarget.8862

**Published:** 2016-04-20

**Authors:** Hadi Tadayyon, Lakshmanan Sannachi, Mehrdad Gangeh, Ali Sadeghi-Naini, William Tran, Maureen E. Trudeau, Kathleen Pritchard, Sonal Ghandi, Sunil Verma, Gregory J. Czarnota

**Affiliations:** ^1^ Physical Sciences, Sunnybrook Research Institute, Sunnybrook Health Sciences Centre, Toronto, ON, Canada; ^2^ Department of Medical Biophysics, Faculty of Medicine, University of Toronto, Toronto, ON, Canada; ^3^ Department of Radiation Oncology, Odette Cancer Centre, Sunnybrook Health Sciences Centre, Toronto, ON, Canada; ^4^ Department of Radiation Oncology, Faculty of Medicine, University of Toronto, Toronto, ON, Canada; ^5^ Division of Medical Oncology, Sunnybrook Health Sciences Centre, University of Toronto, Toronto, ON, Canada

**Keywords:** quantitative ultrasound, tissue characterization, breast cancer, breast cancer chemotherapy, tumor response assessment

## Abstract

**Purpose:**

This study demonstrated the ability of quantitative ultrasound (QUS) parameters in providing an early prediction of tumor response to neoadjuvant chemotherapy (NAC) in patients with locally advanced breast cancer (LABC).

**Methods:**

Using a 6-MHz array transducer, ultrasound radiofrequency (RF) data were collected from 58 LABC patients prior to NAC treatment and at weeks 1, 4, and 8 of their treatment, and prior to surgery. QUS parameters including midband fit (MBF), spectral slope (SS), spectral intercept (SI), spacing among scatterers (SAS), attenuation coefficient estimate (ACE), average scatterer diameter (ASD), and average acoustic concentration (AAC) were determined from the tumor region of interest. Ultrasound data were compared with the ultimate clinical and pathological response of the patient's tumor to treatment and patient recurrence-free survival.

**Results:**

Multi-parameter discriminant analysis using the *κ*-nearest-neighbor classifier demonstrated that the best response classification could be achieved using the combination of MBF, SS, and SAS, with an accuracy of 60 ± 10% at week 1, 77 ± 8% at week 4 and 75 ± 6% at week 8. Furthermore, when the QUS measurements at each time (week) were combined with pre-treatment (week 0) QUS values, the classification accuracies improved (70 ± 9% at week 1, 80 ± 5% at week 4, and 81 ± 6% at week 8). Finally, the multi-parameter QUS model demonstrated a significant difference in survival rates of responding and non-responding patients at weeks 1 and 4 (p=0.035, and 0.027, respectively).

**Conclusion:**

This study demonstrated for the first time, using new parameters tested on relatively large patient cohort and leave-one-out classifier evaluation, that a hybrid QUS biomarker including MBF, SS, and SAS could, with relatively high sensitivity and specificity, detect the response of LABC tumors to NAC as early as after 4 weeks of therapy. The findings of this study also suggested that incorporating pre-treatment QUS parameters of a tumor improved the classification results. This work demonstrated the potential of QUS and machine learning methods for the early assessment of breast tumor response to NAC and providing personalized medicine with regards to the treatment planning of refractory patients.

## INTRODUCTION

Conventional methods of clinical tumor response assessment involve tracking changes in tumor size, using the guidelines provided by Response Evaluation Criteria in Solid Tumors (RECIST) [1]. Such measurements are ascertained using anatomical-based imaging modalities such as X-ray imaging, magnetic resonance imaging (MRI), or conventional diagnostic ultrasound. However, tumor size typically provides late indications of response as measurable changes in tumor size do not occur until several weeks to months after the initiation of the NAC treatment, despite positive response [2]. Currently, no routine clinical imaging is carried out to assess tumor size or response during breast NAC administration in a neoadjuvant setting. Thus, the introduction of a non-invasive functional imaging system that can be used to monitor the early response of a tumor to anticancer therapy can potentially help facilitate personalized treatment for cancer patients, thereby optimizing their therapeutic outcome and recurrence-free survival.

Several imaging methods have been developed in research to assess early therapeutic responses of breast tumors, including diffuse optical spectroscopy (DOS) [3], fluoro-deoxyglucose positron emission tomography (FDG-PET) [4], and diffusion-weighted magnetic resonance imaging (DW-MRI) [5]. Despite a favorable sensitivity in detecting breast tumor response at 4 weeks, DOS has limited tissue penetration depth, thereby limiting its application to superficial tumors. DW-MRI requires substantial capital investment and PET requires the injection of radioactive tracer isotopes, limiting repeated usability and imparting potential long-term health complications. On the other hand, ultrasound is relatively inexpensive and safe and its imaging methods with respect to QUS rely on the inherent changes in tissue microstructure to generate tissue contrast, requiring no external contrast agents.

Quantitative ultrasound (QUS) is a tissue characterization technique which examines the frequency content of the radiofrequency (RF) backscatter ultrasound signals from tissues. According to the theory of ultrasound scattering, the power spectrum of the tissue backscatter signal is affected by parameters such as the size and number density of the ultrasound scatterers. In 1987, Lizzi *et al.* [6] demonstrated that parameters related to the linear regression of the tissue power spectrum are directly linked to the tissue microstructure. These parameters include spectral slope (SS), spectral intercept (SI), and midband fit (MBF). The parameter SS is inversely related to the scatterer size [6], SI is related to scatterer size, scatterer concentration, and the acoustic impedance difference between the scatterer and the background [6], and MBF is related to ultrasound integrated backscatter [7], a measure of the energy efficiency of the acoustic backscatter from a tissue sample [8]. By taking into account differences in tissue microstructure, the aforementioned parameters have enabled the characterization of abnormalities of different tissues such as those in breast, prostate, liver, eye, myocardium, and lymph nodes [9–15]. Alternatively, some studies have found higher-order model derived backscatter coefficient (BSC) parameters such as average scatterer diameter (ASD) and average acoustic concentration (AAC) to be useful in studying tissues, including differentiating mouse models of breast cancer from benign breast masses, grading clinical breast cancer, and detecting cancerous human lymph nodes [10, 15, 16]. Recent pre-clinical studies have demonstrated at high- (>20 MHz) and conventional-(<10MHz) frequency ranges that QUS can be used to detect and quantify tumor cell death *in vivo*, in response to various treatments including photodynamic therapy, radiation therapy, chemotherapy, and anti-vascular therapy [17–20]. Furthermore, pilot clinical studies by Sadeghi-Naini *et al.* [21, 22] has demonstrated the effectiveness of QUS and texture analysis methods in the assessment of patients' breast tumor responses to NAC as early as 1 week into their several-month treatment. In those studies, Sadeghi-Naini *et al*. posited that at a clinically relevant frequency range (<10 MHz), spectral parameters such as MBF, SI, and SS are sensitive to changes in tumor microstructure which occur as a result of therapeutic effects, and therefore can correlate to early signatures of tumor response. Furthermore, statistical texture analysis of the QUS images using the gray-level co-occurrence matrices (GLCM) considering the heterogeneity of the tumor response was suggested to improve the discrimination of responsive patients from non-responsive ones [22]. However, those studies were limited to only statistical significance tests and the use of a simple classifier (Fisher linear discriminant (FLD)) applied on a small patient database (N=25) and performance measures were obtained without cross-validation (training and testing sets were identical), resulting in over-optimistic values. More recently, Tadayyon *et al*. [23] compared tumor response prediction sensitivity and the specificity of the MBF when the power spectrum was corrected for attenuation and vice versa. They demonstrated that estimating the acoustic attenuation of the patient's tumor and correcting the power spectra accordingly, not previously done [21, 22], increased the sensitivity of MBF to response detection by 12% and specificity by 17%.

Here, we propose a new and improved approach for the QUS prediction of breast tumor response to neoadjuvant chemotherapy. Specifically, we have made the following five improvements compared to similar previous works [22, 24]:

1. We have used the largest population size (N = 58) studied to date on QUS characterization of LABC tumor response to NAC, which is double the size of the most recent study [24]. This represents over 200 volumetric ultrasound scans of patient tumours.
2. We have included new QUS features not investigated previously for this application, including attenuation coefficient estimate (ACE), attenuation-corrected QUS features, as well as spacing among scatterers (SAS).
3. We have performed leave-one-patient-out cross-validation in order to evaluate the performance of the classifier when subjected to unseen data.
4. We have used a new classifier, the KNN classifier, to perform discriminant analysis. Since the search radius can be tuned, the KNN classifier can learn the local structure of a feature space more effectively than a linear classifier. This is important especially in a tumor response classification task such as this, since tumor response is not discrete but rather heterogeneous, with many variations in degrees of response.
5. The study here found, for the first time, that including pre-treatment QUS features in the QUS model improves the discrimination of response.

## RESULTS

Patient characteristics, including age, initial tumor size, tumor subtypes, and bulk tumor shrinkage for responders and non-responders are summarized in Table 1. All patients were females aged between 29 and 67 years with a mean age of 49 years. Tumor size ranged from 2 to 13 cm, with a mean size of 6.3 cm. The tumors were mainly of the invasive ductal carcinoma type not otherwise specified (90% of cases). The remaining 10% of cases were comprised of invasive lobular carcinoma (5%) and other types of breast cancer (5%). The ultimate clinical response rate to NAC in the sample population was 72% and responders demonstrated a mean tumor shrinkage of 68 ± 47% whereas the non-responders demonstrated mean bulk shrinkage of −16 ± 57%. Bulk tumor shrinkage was defined as the relative reduction in the sum of tumor diameters from pre-treatment to pre-operation. Size measurements were ascertained using breast DCE-MRI obtained at these two times. Detailed individual patient characteristics and responses are provided in Tables A.1 and A.2.

**Table 1:** Summary of patient characteristics

Parameter	Mean +/− SD / count		
Age (y)		49 ± 10	
Pre-tx tumor size (cm)		6.3 ± 3.2	
	No.		%
Tumor subtype			
IDC	52		90
ILC	3		5
Other	3		5
Responders	42		72
BTS (%)		68 ± 47	
Non-responders	16		28
BTS (%)		−16 ± 57	

IDC = invasive ductal carcinoma, ILC = Invasive lobular carcinoma, BTS = bulk tumor shrinkage (percent change in tumor size)

± indicates standard deviation

**Table A1:** Patient characteristics and treatment information

Pt No.	Age	Initial tumor Dimensions (AP × ML × SI cm)	Histology	Grade	ER	PR	HER2	Treatment
1	55	5.4 × 5.0 × 2.3	IDC	I	-	+	+	FEC and Taxol, Herceptin
2	53	7.4 × 7.0	IDC with mucinous features	I	+	+	-	Epi and Taxotere
3	41	5.3 × 4.4 × 4.7	IDC	II	+	+	+	Docetaxel, carboplatin, trastuzumab
4	65	10.0 × 10.0	IDC	I	-	-	-	AC & Taxotere
5	50	4.0 × 5.0	IDC	III	+	+	+	AC + docetaxel, trastuzumab
6	33	3.0 × 3.0	IDC	I	+	+	-	AC & Taxol
7	33	5.4.0 × 5.0 × 8.0	IDC	II	+	+	+	AC + docetaxel, paclitaxel, trastuzumab
8	48	4.9.0 × 4.9.0 × 4.1.0	IDC	III	+	+	-	AC + docetaxel
9	36	4.4 × 3.9 × 5.8	IDC	II	+	+	-	AC + paclitaxel
10	40	4.4 × 3.4	IDC	III	-	-	-	AC + paclitaxel
11	62	12.0 × 14.0	IDC	II-III	-	-	-	FEC + docetaxel
12	59	6 × 2.3 × 4.3	IDC	III	-	-	-	AC + paclitaxel
13	53	8.4 × 9.4 × 12.7	Metaplastic carcinoma	III	-	-	-	AC + cisplatinum, gemcitabine platinum
14	48	7 × 9.0	IDC	II	+	+	+	AC-Taxol and Herceptin
15	50	13.0 × 11.0	IDC	III	-	-	-	AC + paclitaxel
16	49	7.1 × 5.5 × 8.9	IDC	III	-	-	+	Docetaxel, trastuzumab
17	40	3 × 2.4 × 3.0	IDC	III	+	+	+	AC + paclitaxel, trastuzumab
18	56	2.4 × 2.7 × 3.2	IDC	II	-	-	+	AC + paclitaxel, trastuzumab
19	49	2.4 × 2.8 × 1.4	IDC	II	-	-	+	AC-Taxol and Herceptin
20	47	5.2 × 4.0 × 4.0	IDC	NA	+	+	-	FEC + docetaxel
21	52	4.1 × 3.0 × 2.5	IDC	NA	+	+	-	AC + docetaxel, paclitaxel
22	44	9.9 × 4.5 × 9.7	IDC	III	+	+	+	AC + paclitaxel, trastuzumab
23	38	9.0 × 6.6 × 6.0	IDC	II*	+	+	-	AC + paclitaxel
24	58	1.9 × 1.4 × 1.6	IDC with basal like features	III	-	-	-	AC + paclitaxel
25	35	5.9	IDC	III	-	-	-	AC-Taxol
26	38	8.0 × 8.0	IDC	III	-	-	+	Dose-dense AC + paclitaxel, trastuzumab
27	47	8.0 × 10.0	IDC	II	+	+	-	Dose-dense AC + paclitaxel
28	57	7.9 × 4.1 × 5.5	IDC	III	-	-	-	Dose-dense AC + paclitaxel
29	47	6.3 × 4.1 × 7.4	IDC	NA	-	-	+	Dose-dense AC + paclitaxel, trastuzumab
30	55	6.6 × 12.8 × 6.8	IDC	II	+	+	-	AC + paclitaxel
31	32	6.0 × 7.0 × 3.0	IMC	Ϯ	+	+	+	AC + paclitaxel + Herceptin
32	38	2.3 × 2.5 × 2.5 & 1.0 × 1.0 × 0.7	IDC	III	-	-	-	AC + paclitaxel
33	45	6.5 × 5.0	IDC	I	+	+	+	AC-Taxol + Herceptin
34	55	10 × 5 × 10.5	IDC	III	-	-	-	dose dense AC + taxol
35	59	8.0 × 5.7 x3.0	IDc	II	+	+	+	FEC + docetaxel, trastuzumab
36	37	2.5 × 2.0	IDC	III	+	+	-	dose dense AC + taxol
37	50	9.0 × 7.0 × 3.0	IDc	II	+	+	-	AC + paclitaxel
38	54	3.6 × 3.6 × 2.3	IDC	NA	+	-	-	TC
39	55	1.6 × 1.2	ILC @ 12H; IDC @ 2H	NA	+	+	-	FEC-D
40	50	7.3 × 2.5 × 7.3	IDC	III	-	-	-	FEC-D
41	55	3.3 × 3.4 × 3.4	IDC	III	-	-	-	TC
42	44	3.0 × 3.5 × 1.5	IDC with prominent lymphoid stroma	III	-	-	-	FEC-D
43	60	8.7 × 9.0 × 5.2	Invasive lobular carcinoma	NA	+	-	-	FEC-D
44	64	6.4 × 3.2 × 8.7	ILC(Invasive lobular carcinoma	II	+	+	-	FEC-D
45	67	3.2 × 8.7	IDC	II	-	-	-	FEC-D
46	52	2.6 × 1.2 × 1.6	IDC	II	-	-	-	FEC-D
47	47	8.0 × 7.0	IDC	III	-	-	-	FEC-D
48	56	10.0 × 10.0	IDC	II	+	+	+	Paclitaxel and Herceptin
49	45	2.3 × 2.0	IDC	NA	+	+	+	FEC-DH.
50	59	4.9 × 2.1 × 1.4	IDC	II	+	+	-	FEC-D
51	66	3.5 × 5.2 × 2.1	IDC	II-III	+	+	+	TCH
52	49	1.8 × 2.1 × 2.1	IDC	I	+	+	+	dose dense AC + taxol + Herceptin
53	39	6.3	Invasive carcinoma with ductal & lobular features	II*	+	+	-	FEC-D
54	62	4.4 × 6.3 × 3.3	IDC	III	-	-	-	dose dense AC/taxol
55	58	5.2 × 5.2 × 4.4	IDC	I	+	+	+	dose dense AC/Taxol+Herceptin
56	58	2.3 × 4.0 × 2.3;1.6 × 1.8 × 1.6	IDC	III	-	-	+	Taxotere/Carboplatin and Trastuzumab (TCH)
57	45	2.7 × 3.2 × 2.0	IDC	III	+	+	-	ACT
58	29	4.2 × 2.9 × 2.7	IDC	III	+	+	-	dose dense AC paclitaxol

ML = medial-lateral, SI = superior-inferior, and AP = anterior-posterior

Ϯ tubular formation =3/3, nuclear pleomorphism = 2/3, mitotic score could not be determined since tumor was present in small clusters

* indicates that the information was obtained from mastectomy report and not the biopsy report, as this was the only available source of this information.

**Table A2:** Patient responses to administered regimens according to RECIST and cellularity

Pt No.	Residual tumor size	BTS (%)	Notes	Response
1	No residual	100		Responder
2	7 × 5 × 3	5	Very low cellularity	Responder
3	2.7 × 2.5 × 2.4	49	Very high cellularity	Non-Responder
4	1.6 × 0.8 × 0.5	84		Responder
5	No residual	100		Responder
6	1.4	53		Responder
7	No residual	100		Responder
8	1.4 × 1 × 1	71		Responder
9	11.4	−97		Non-Responder
10	No residual	100		Responder
11	No residual	100		Responder
12	2.6 × 2.5 × 2.5	57		Responder
13	whole breast	0		Non-Responder
14	5	44	Patient switched therapy after 1 cycle due to poor response to first therapy	Non-Responder
15	4	69		Responder
16	No residual	100		Responder
17	No residual	100		Responder
18	0.2 × 0.2	93		Responder
19	1.4 × 2.4 × 1.4	96		Responder
20	6.5	−25		Non-Responder
21	No residual	100		Responder
22	2 × 1 × 1 + 1.6 × 1 × 0.5	64		Responder
23	2.9 × 2 × 1.5 + 2 × 1.5 × 1	46		Responder
24	No residual	100		Responder
25	No residual	100		Responder
26	No residual	100		Responder
27	12.5 × 4.5 × 3.5	−25		Non-Responder
28	No residual	100		Responder
29	No residual	100		Responder
30	17	−33	Very low cellularity	Responder
31	7.4	−6	bed of scattered microscopic cancer foci	Responder
32	2.8 × 3 × 2.3 + 1.5 × 1.6 × 1.1	−31		Non-Responder
33	2.8 + 2	26		Non-Responder
34	No residual	92		Responder
35	No residual	100		Responder
36	2.2 × 1.5 × 1.1	12		Non-Responder
37	1.2	87		Responder
38	5.5	−139	Scattered tumor clusters	Responder
39	1.2 × 0.9 × 0.7	25		Non-Responder
40	2.1	71		Responder
41	1.8	47		Responder
42	No residual	100		Responder
43	8.0 × 5.0 × 4.5 + 3.0 × 2.5 × 1.7	−22		Non-Responder
44	19	−197		Non-Responder
45	3.2 × 3 × 1.8	63		Responder
46	2.5 × 0.4 × 0.4	4	Very low cellularity	Responder
47	4.5 × 3.1 × 2.9	44		Responder
48	8.4 × 5.1 × 2.8	16	Very low cellularity	Responder
49	No residual	100		Responder
50	2.8 × 2.5 × 1.5	43		Responder
51	4 × 3	23		Non-Responder
52	No residual	100		Responder
53	1.7×1.5×1	37		Responder
54	12.6 × 6 × 3	−100		Non-Responder
55	3.4	35		Responder
56	No residual	100		Responder
57	3	6		Non-Responder
58	4	0		Non-Responder

**Figure 1:**
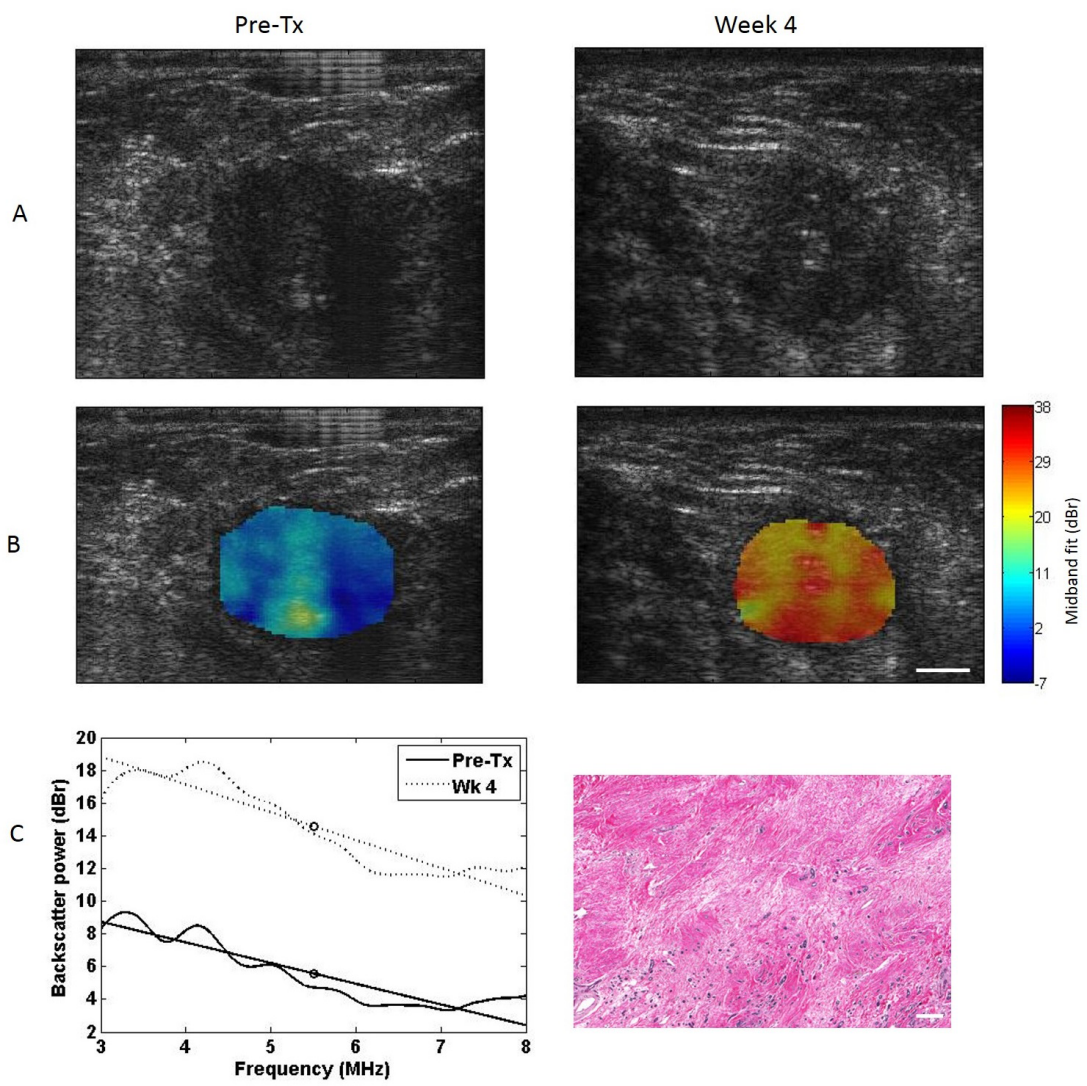
Representative data for a responding patient B-mode images **A.** MBF images **B.** and power spectra **C left.** before and 4 weeks after the start of chemotherapy treatment. Hematoxylin and eosin histology histology image post–surgery **C right.** Data in the left column represent pre-treatment data, obtained prior to treatment initiation, and data in the right column represent week 4 data. US scale bar represents 1 cm, histology scale bar represents 100 μm.

**Figure 2:**
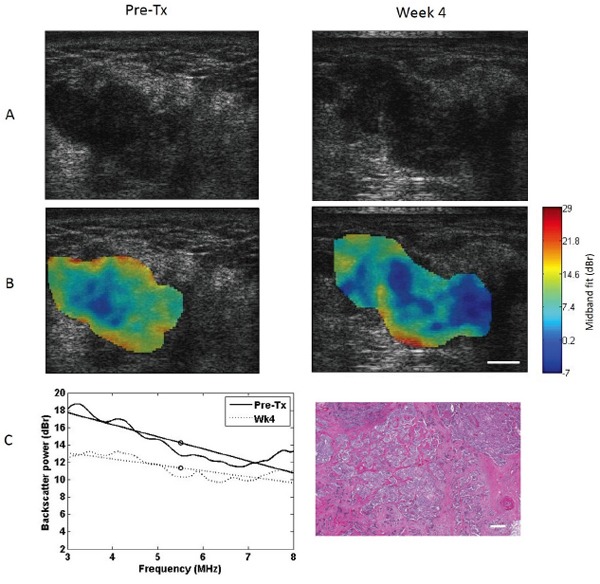
Representative data for a non-responding patient B-mode images **A. MBF images B.** and power spectra **C left.**before and 4 weeks after the start of chemotherapy treatment. Hematoxylin and eosin histology histology image post–surgery **C right.** Data in the left column represent pre-treatment data, obtained prior to treatment initiation, and data in the right column represent week 4 data. US scale bar represents 1 cm, histology scale bar represents 100 μm.

Representative images of a responding breast tumor and a non-responding breast tumor before treatment initiation and 4 weeks after treatment initiation (1-2 cycles of NAC) are presented in Figures 1 and 2. For each tumor, B-mode images, MBF images overlaid on the B-modes, power spectra before and 4 weeks after the start of treatment, and magnified hematoxylin and eosin (H&E) stained histology sections of whole-mount breast specimens obtained post-surgery (mastectomy/lumpectomy) are shown. These data were selected for illustration as MBF was a parameter, which demonstrated statistically significant changes at early weeks (1 and 4). Whereas B-mode images showed no appreciable changes in the tumor 4 weeks into treatment, a marked increase in MBF could be observed in the responding tumor region as a result of 4 weeks of NAC (1-2 cycles). The non-responding tumor, on the other hand, demonstrated no change or decrease in MBF. The before/after superimposed power spectra demonstrated the same concept graphically, where MBF is marked by a circle in the middle of the regression line (Figure 1 and 2 (C left)). The histology image of the responding tumor indicates a stroma-filled tissue (pink staining) with small isolated patches of glands (purple staining), demonstrating therapeutic effects. On the other hand, the histology of the non-responding tumor shows a gland-dominated tumor with low stromal collagen density, indicating little to no therapeutic effect.

**Figure 3:**
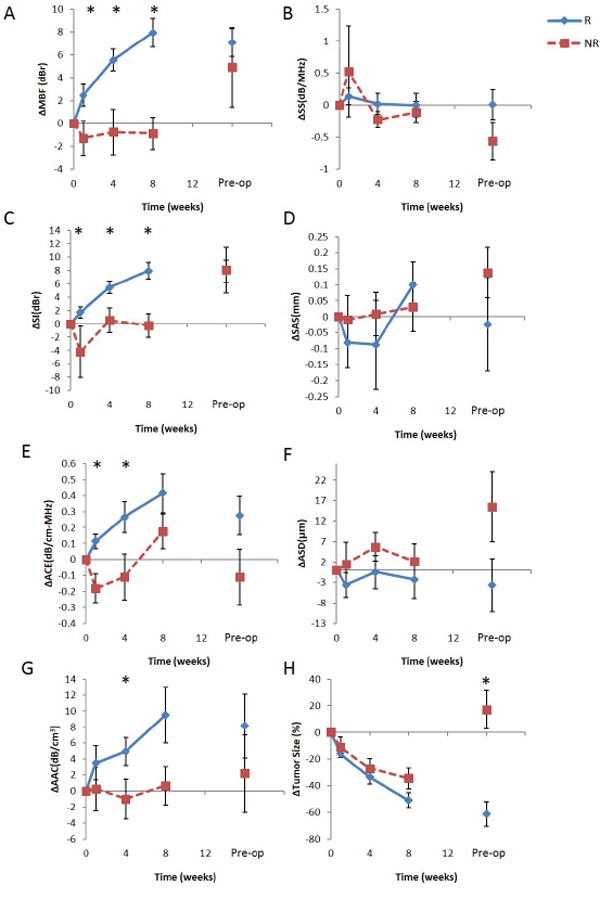
Comparison between QUS parameters A-G. and the RECIST-based tumor size reduction H. for tracking patient tumors during chemotherapy QUS and RECIST values were averaged over responder (blue diamond) and non-responder (red square) groups, and plotted over the treatment time. Patients were grouped based on their pathological clinical response determined post-chemotherapy. All values were normalized to week 0 by subtraction. Error bars represent standard error of the mean.

Figure 3 compares QUS parameters with the RECIST metric for tracking changes in the tumor during NAC. Average QUS data obtained from responding and non-responding groups are plotted versus treatment time in Figure 3A-3G. Patients were grouped based on their ultimate clinical/pathological responses. The vertical axes represent the absolute difference in QUS parameters relative to week 0 (pre-treatment), which is denoted by a Δ prefix. For instance, ΔMBF at week 4 is computed as MBF(week 4) – MBF(week 0). Parameters related to the intensity of the frequency-dependent backscatter (i.e. ΔMBF, ΔSI, ΔAAC) demonstrated, on average, an increase with treatment time for responders. Based on unpaired *t*-test comparison of responder and non-responder groups, right-tailed with 95% confidence, this increase was statistically significant at weeks 1, 4, and 8 for ΔMBF (p = 0.042, <0.005, <0.005, respectively), and at weeks 1, 4, and 8 for ΔSI (p = 0.034, 0.010, <0.005, respectively). Patients in the responding group demonstrated a greater increase in ΔACE compared to non-responders, which were statistically significant at weeks 1 and 4 (p <0.005 and 0.042, respectively). On the other hand, ΔSS, ΔASD, and ΔSAS values did not show any significant changes between responders and non-responders at any time during the treatment. As expected, the mean tumor size reduction shown in Figure 3H was not significantly different in responders versus non-responders at any time (*p* = 0.89, 0.53, and 0.42 at week 1, 4, and 8, respectively) except at the end of the several-month treatment (*p* = 0.0011 at pre-op). Whereas a 30% mean size reduction occurred in responders at week 4 (Figure 3H), non-responders also had a mean reduction of almost 30% at week 4, and the difference between the groups was not statistically significant. After week 4, whereas responding tumors continued shrinking, non-responding tumors grew to 20% larger than their original size between week 8 and pre-op, which had approximately an 8 to 10 week gap.

In order to compare the effectiveness of different QUS parameters in differentiating responding tumors from non-responding ones, the KNN algorithm was run for each QUS parameter separately and classification accuracy was computed. Table 2 summarizes the performance of individual QUS parameters in predicting response in terms of classification accuracy and statistical significance (p-value) for weeks 1, 4, and 8. The classification results are based on a 2-neighbor search area and using the Euclidean distance metric, which provided the optimal classification. The results demonstrated that the MBF parameter was most effective in response detection at all weeks (accuracy = 61 ± 8%, 65 ± 5%, and 85 ± 5%, for weeks 1, 4, and 8, respectively), followed by SI (accuracy = 55 ± 8%, 65 ± 11%, 74 ± 6%, respectively). Overall, performances improved at week 8 compared to those of weeks 4 and 1.

**Table 2:** *p*-values and classification performances (accuracy) of individual QUS parameters for differentiating responders from non-responders at weeks 1, 4 and 8

		Week 1	Week 4	Week 8
ΔMBF	Accuracy	61 ± 8	65 ± 5	85 ± 5
	p-value	0.042	<0.005	<0.005
ΔSI	Accuracy	55 ± 8	65 ± 11	74 ± 6
	p-value	0.034	0.010	<0.005
ΔACE	Accuracy	54 ± 8	64 ± 4	60 ± 10
	p-value	<0.005	0.042	0.259
ΔAAC	Accuracy	58 ± 8	64 ± 9	69 ± 8
	p-value	0.404	0.069	0.135
ΔSS	Accuracy	53 ± 8	62 ± 7	63 ± 15
	p-value	0.424	0.368	0.727
ΔASD	Accuracy	54 ± 9	61 ± 9	60 ± 13
	p-value	0.382	0.377	0.570
ΔSAS	Accuracy	62 ± 7	58 ± 8	69 ± 7
	p-value	0.606	0.678	0.590

Parameters are sorted from highest to lowest accuracy at week 4. The bold entry indicates the best performance. Reported values are mean and standard deviation of the accuracies in percentages. Results were obtained by running the classification 10 times using 10 random samples from responders group

Table 3 presents the RECIST-based versus multiparameter-QUS-based patient response classification results. Sensitivity was defined as the ratio of the number of true responders to total number of responders (expressed as a percentage). Specificity was defined as the ratio of the number of true non-responders to the total number of non-responders in percentage. Accuracy was determined as the percentage of total number of correctly classified patients to the total number of patients. The first row presents the RECIST-based response classification, which was performed by classifying each patient based on 30% reduction at each follow-up visit and comparing the prediction with their “true” response, assumed to be the ultimate clinical/pathological response. The second row presents the classification performance obtained using only changes in QUS features relative to pre-treatment, whereas the third row presents the classification performance obtained using pre-treatment QUS features, and the combination of pre-treatment and changes in QUS features during treatment. The fourth row presents the *p*-values of the significance of the difference between the accuracies of the second and third rows. An asterisk indicates a significant difference. Leave-one-patient-out cross-validation was performed on the KNN classifier to obtain the overall sensitivity, specificity, and accuracy values. All possible combinations of the 7 QUS parameters (ΔMBF, ΔSS, ΔSI, ΔSAS, ΔACE, ΔASD, ΔAAC) were investigated for feature selection. As expected, the RECIST method showed the poorest discrimination between responders and non-responders at all times during the treatment (accuracies of 30%, 52%, and 68% at weeks 1, 4, and 8, respectively, as presented in Table 3, row 1). On the other hand, the QUS-based model including the optimal parameter combination of [ΔMBF ΔSS ΔSAS] displayed promising accuracies (accuracy = 60 ± 10%, 77 ± 8%, 75 ± 6%, at weeks 1, 4, and 8, respectively, as presented in Table 3, row 2). Combinations of 4 or more parameters have not been reported since no improvement was observed beyond 3 parameters. A separate feature selection was performed for the case when the pre-treatment values were included. Furthermore, the QUS biomarker consisting of [MBF_wk0_ ΔMBF SS_wk0_ ΔSS SAS_wk0_ ΔSAS] differentiated responders from non-responders with improved accuracies of 70 ± 9% 80 ± 5%, and 81 ± 6% at weeks 1, 4, and 8, respectively, as presented in Table 3, row 3. The inclusion of pre-treatment information demonstrated a 10% improvement in the accuracy at week 1 which was also statistically significant (p-value = 0.03). Even at baseline (pre-treatment), the response of the patients could be predicted with 65 ± 9% accuracy using the set [MBF_wk0_ SS_wk0_ SAS_wk0_].

**Table 3:** A comparison of the response classification results obtained using tumor size alone (RECIST criteria), using KNN-based QUS feature combination, and using KNN-based QUS feature combinations with the addition of pretreatment data. Reported are sensitivity (Sen), specificity (Spe), and accuracy (Acc) mean ± standard deviation

	Pre-Tx			Week 1			Week 4		
	Sen	Spe	Acc	Sen	Spe	Acc	Sen	Spe	Acc
RECIST		NA		16	60	30	53	50	52
ΔQUS		NA		61 ± 13	59 ± 9	60 ± 10	79 ± 10	76 ± 11	77 ± 8
ΔQUS + QUSw_0_	67 ± 13	63 ± 7	65 ± 9	76 ± 11	64 ± 11	70 ± 9	80 ± 9	79 ± 5	80 ± 5
p-value		NA			0.03*			0.33	

The results (sensitivity, specificity, and accuracy in percentages) are reported for weeks 1, 4 and 8 obtained from leave-one-out analysis cross-validation. ΔQUS represents [ΔMBF ΔSS ΔSAS] and QUS_w0_ represents [MBF_w0_ SS_w0_ SAS_w0_]. The last row presents the p-value significance of the difference between the mean accuracies of ΔQUS and ΔQUS + QUS_w0_ KNN models. Reported values are mean and standard deviation of the accuracies obtained by running the classification 10 times using 10 bootstrap samples from responder group.

**Figure 4:**
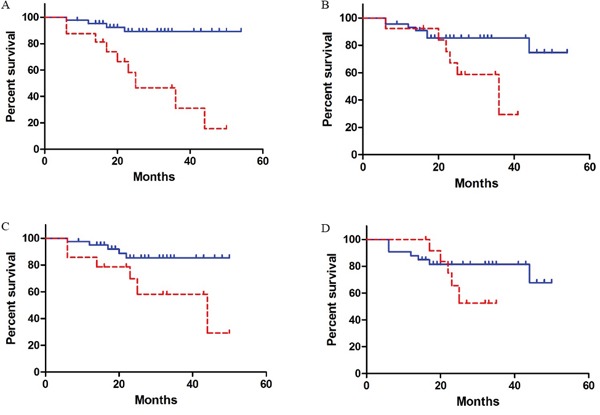
Kaplan-Meier survival curves for responding (solid line) and non-responding (dashed line) patients Patients were classified based on clinical/histopathological information. **A.** Patients were classified based on the QUS biomarkers (including week 0 data) obtained at weeks 1, 4, and 8, respectively **B, C, D.**

In order to compare the predictions of QUS and histopathology on the recurrence free survival (RFS) of the patients, Kaplan-Meier survival analysis was performed and the results are presented in Figure 4. The median follow-up time was 25 months. The RFS curves were divided into responder and non-responder groups. A log-rank test was performed to compare the RFS rates between the responders and non-responders [25]. The RFS curves obtained from QUS biomarkers demonstrated statistically significant differences between the response groups at weeks 1 and 4 (log-rank *p*-value = 0.035, 0.027, respectively) as did the RFS curves obtained from histopathology information (log-rank *p*-value = 0.0002). However, RFS curves obtained from QUS biomarkers at week 8 did not show significant difference between the response groups (log-rank *p*-value = 0.26).

## DISCUSSION

This study demonstrated, for the first time, using a relatively large patient database and using a leave-one-patient out classifier evaluation that multi-parametric QUS applied at a clinically relevant frequency range (<10 MHz) can be used to non-invasively predict breast tumor response to NAC as early as after 1-2 cycles (1-4 weeks) with reasonable accuracy (80%), whereas RECIST-based tumor size change is only 52% accurate in predicting response at week 4 with a 30% threshold. Additionally, RFS analyses performed demonstrated that when the ultrasound biomarkers [MBF, SS, SAS], which include pre-treatment values along with the change at a specified time during treatment, were used to predict the RFS, responder and non-responder RFS rates were statistically significantly different when classifying patients based on data at weeks 1 and 4. Although the results of this study were not used to modify the treatments of the patients, the findings suggest that ultrasound biomarkers can predict the RFS rates of responding and non-responding patients within weeks almost as accurately as patient ultimate clinical response based on clinical and histopathology information obtained many months later. The reason for the poor separation of the RFS rates of the groups predicted from week 8 QUS data was likely due to the QUS biomarker being sensitive only to early microstructural changes in the tumor during treatment. Post-surgical histology images demonstrated a considerable extent of fibrosis potentially mixed with cell death in the responding tumor bed. Thus, it is posited that at 8 weeks, the beginning of fibrotic changes contributed to altering the QUS measurement of cell death.

Previously developed theories about ultrasound detection of cell death support the findings in this study. Just as the parameters related to the backscatter intensity and acoustic concentration (i.e. MBF and SI) increased in tumors undergoing cell death *in vivo* in previous studies [19], MBF, SI, and AAC values increased in clinically responding tumors in this study. Classification results determined that MBF was the most effective parameter in the discrimination of responders from non-responders at weeks 4 and 8 (Table 2). This suggests that response-related changes in a tumor are linked to the energy efficiency of acoustic backscatter from the tumor tissue. Since changes in ACE obtained at weeks 1 and 4 were statistically significant in responders compared to those of non-responders (p = 0.004 and 0.039, respectively), it is likely that the attenuation correction of the tumor spectra helped in accentuating the MBF parameter to response detection. Furthermore, the MBF changes in the responding-tumor patient population became highly statistically different from those of non-responding tumors at week 8 (p < 0.005). The increase in the ACE observed in responding tumors over treatment time was concordant with the increase in attenuation coefficient with cell death extent observed in previous high-frequency QUS cell treatment characterization studies [26].

Classification results using the multiparametric QUS model demonstrated that increasing the number of QUS parameters submitted to the classification system improved the discrimination power, but not beyond three parameters. Whereas previous studies found classical parameters (MBF and SI) to be sensitive to detecting breast tumor response at 4 weeks, we found that MBF, SI, ACE, and AAC all have comparable accuracies in predicting tumor response (65%, 65%, 64%, and 64% respectively at week 4). Combining the changes and pre-treatment values of MBF, SS, and SAS provided the best prediction of response (70 ± 9% at week 1, 80 ± 5% at week 4, and 81 ± 6 and week 8). This may be due to structural tumor properties before NAC being linked to tumor aggressiveness and consequently the likelihood of response to NAC.

In terms of statistical analysis, the ΔMBF, ΔSI, and ΔACE parameters in our study demonstrated a significant change in responders at week 1 (p < 0.05), just as tracer uptake change did after one cycle in the PET study (p < 0.05) [4], just as total diffusion change did after one cycle of NAC in the DW-MRI study (p < 0.05) [5], and just as changes in deoxygenated hemoglobin, oxygenated hemoglobin, total hemoglobin concentration, water percentage, and tissue optical index did at week 1 in the DOS study (p < 0.05) [3]. Recently, a genetic method of monitoring metastatic breast cancer has been proposed, demonstrating circulating tumor DNA as an effective biomarker for this purpose [27]. However, this method is invasive in its nature and time consuming, as it involves many steps including blood sample centrifugation, DNA extraction, polymerase-chain-reaction to detect genomic mutations, and assay of circulating tumor cells. On the other hand, the ultrasound-based method here permits breast ultrasound imaging and response assessment to be performed using one system and in one session and does not rely on tumor-specific genetic markers. It is sensitive to the biophysical changes which accompany cell death – the induction of which is the goal of cancer chemotherapy. Evidence demonstrates that patients who respond well to chemotherapy may benefit from longer regimens of efficacious chemotherapy and suggest that ineffective treatments should be changed [28].

Currently the standard of care for patients receiving NAC only includes pre-treatment and post-treatment imaging, using typically DCE-MRI, but does not routinely include intra-treatment imaging for tumor size assessment. Furthermore, ultrasound imaging is not reliable for tumor size measurement due to attenuation artefacts which cast shadows on the distal end of deep-set tumors. However, this had minimal effect on QUS assessment in this study, since the ROIs were selected in the center of the tumor (∼ 90% coverage), avoiding regions of artefacts. Although intra-treatment tumor size was recorded in this study as measured by the physician during follow-up physical examinations, this method has limited reproducibility since measurements were made by different physicians. Thus, measurements reported here should be assumed approximate.

As demonstrated by the results, whereas ΔMBF was the most effective single QUS parameter for classifying patient response, combining ΔMBF with ΔSS and ΔSAS improved the classification response accuracy at week 4 from 65 ± 5% to 77 ± 8%. A point of note is that ΔSS (week 0 and 4) and ΔSAS (week 0 and 4) were less accurate than other parameters investigated such as ΔSI, ΔACE, ΔAAC, and ΔASD, according to the results in Table 2. However, since ΔSS and ΔSAS are features that are independent from ΔMBF, the discriminating power increased when these parameters were combined. Particularly, MBF describes the acoustic concentration, SS describes the size of the scatterers, and SAS describes the distance between regularly-spaced scatterers.

The QUS results obtained indicated poor separation between responders and non-responders at the pre-operative scan time. This is expected and likely due to the large time gap between the end of neoadjuvant treatment and surgery (usually several weeks), where minimal or no cell death had occurred at the time of data acquisition. Additionally, tumor ROI selection in pre-operative images was difficult in complete pathologic responders who had no residual tumor, and were therefore excluded from the analysis. As expected the early investigated times at weeks 1 and 4 indicated the best separation between responders and non-responders. These were selected to span cycles of NAC and it remains unknown if other times sooner or later would be useful for analyses. Despite this, the sensitivity and specificity and consequent accuracy were significant for predicting ultimate patient clinical response.

## CONCLUSIONS

In summary, this study demonstrated for the first time, using a relatively large patient cohort and leave-one-out classifier evaluation, that the hybrid QUS biomarker [ΔMBF, ΔSS, ΔSAS] can, with good sensitivity and specificity, detect the response of LABC tumors to NAC as early as after 1 cycle (1 week) of administration. Extending efficacious treatments and switching ineffective ones early based on indications of QUS biomarkers may likely result in improved RFS. The findings of this study also provided insight into pre-treatment ultrasonic scattering properties of a tumor potentially contributing to a prediction about its therapeutic resistance before the initiation of therapy.

## MATERIALS AND METHODS

### Ultrasound data acquisition and processing

This prospective study was reviewed and approved by the institution's research ethics board. After obtaining informed consent, ultrasound RF data were collected from the affected breast of patients (*N* = 58) with locally advanced breast cancer (LABC) prior to NAC treatment initiation and at four times during the course of the treatment - weeks 1, 4, 8, and prior to surgery (mastectomy/lumpectomy). Patients recently diagnosed with locally advanced invasive breast cancer within one week, including invasive ductal carcinoma, invasive lobular carcinoma, and other forms of invasive cancer, including all grades, were referred from the diagnostic clinic to our study. This included patients with tumors larger than 5 cm and/or tumors with locoregional lymph node, skin, and chest wall involvement as per guidelines reported in [29]. All clinical and ultrasound data obtained for this study were dated back to patients treated between January 2009 and August 2013. Treatment regimens varied from 5-fluorouracil, epirubicin and cyclophosphamide followed by docetaxol (FEC-D), to Adriamycin followed by paclitaxel (AC-T), or taxol followed by herceptin varying from weekly to tri-weekly cycles. Individual patient treatment regimens are provided in Table A.1.

**Figure 5:**
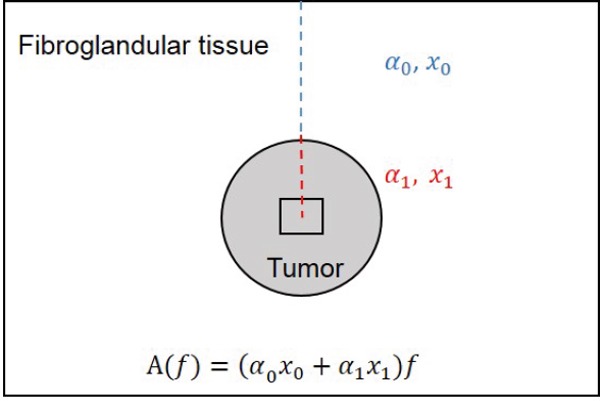
A diagram depicting the ultrasound attenuation correction procedure *α*_0_ and *x*_0_ are the attenuation coefficient (assumed to be 1 dB/cm-MHz) and length of the intervening tissue above the ROI, respectively. *α*_1_ and *x*_1_ are the local attenuation coefficient estimate (ACE) of the ROI and the distance from the top of the ROI to the center of RF block. *A(f)* is the total attenuation from the top of the image to the center of the RF block.

Breast ultrasound data were collected by an experienced sonographer using a clinical scanner (Sonix RP, Ultrasonix, Vancouver, Canada) employing a 6 MHz center frequency linear array transducer (L14-5-60), sampling at a rate of 40 MHz, with the focus set at the midline of the tumor and maximum imaging depth set to 4-6 cm, depending on tumor size and location. Standard B-mode imaging was used for anatomical navigation, and acquisition volume was determined based on the tumor location reported in biopsy findings. Approximately 3-5 image planes were acquired from the tumor, depending on the tumor size. Regions of interest (ROI) were contoured around the tumor within each image plane and segmented into smaller blocks, called RF blocks. The ROI was then divided into 2 by 2 mm blocks, with adjacent overlap of 80% in both axial and lateral directions. A 2 by 2 mm RF block corresponds to 10 spatial pulse lengths axially and 5.5 beamwidths laterally, which meets the minimum ROI size requirements for obtaining reliable scatterer property estimates [30, 31]. A normalized power spectrum was then computed for each RF block using a phantom reference, and was corrected for the total attenuation, *A(f)*, from the top of the image down to the center of the RF block, as illustrated in Figure 5. The total attenuation consisted of two components: attenuation of the intervening tissue, *α*_0_, assumed to be 1 dB/cm-MHz based on reported ultrasound tomography measurements of the breast [32], and the local attenuation estimate of the ROI, *α*_1_, which is also referred to here as ACE and is estimated using the spectral difference method [33]. After obtaining attenuation corrected normalized power spectra from all RF blocks within the ROI, a parametric image was computed for each QUS parameter. The QUS parameters investigated were MBF, SS, SI, SAS, ACE, ASD, and AAC. More details about QUS analysis are provided in the appendix. Since spectral normalization was performed using a homogeneous tissue-mimicking phantom prior to parameter estimation, effects of tumor depth and size were minimized. In addition to acquiring ultrasound data, tumor sizes reported by oncologists by physical examination in the follow-up visits were also examined. Size reports were corroborated by ultrasound imaging results but clinical physical examination documentation was used for tumor size measures.

### Classification and statistical analyses

All QUS results were compared with the clinical standard response of each patient, determined based on the RECIST guideline. This was determined at the end of the patient's several-month treatment by measuring the reduction in gross tumor size based on dynamic contrast enhanced magnetic resonance images (DCE-MRI) cross-verified with whole-mount breast histopathology obtained post-operatively. Since the focus of this study was a binary classification of response, the standard four categories of response defined in the RECIST guideline were merged into two categories by grouping complete and partial responses into “response” and grouping stable and progressive disease responses into “non-response”. A recent study demonstrated that residual tumor cellularity is an important prognostic factor in breast cancer neoadjuvant treatment, which should be taken into account in conjunction with the RECIST metric of bulk tumor shrinkage (BTS) [34]. Accordingly in this study, a patient was deemed to be a clinical responder if the sum of the lengths of the tumor foci was reduced by more than 30% or if in the non-mass enhancing area, the pathologically determined residual tumor cellularity was low. Conversely, a patient was considered a clinical non-responder if the sum of the lengths of their tumor foci was reduced by less than 30% or the residual tumor cellularity remained high. In cases (infrequent) where the RECIST-based response conflicted with the pathological response, the pathological response was used to determine the true response.

The mean changes in each QUS parameter were compared between the clinical responder and non-responder groups at each time. Initially, a Shapiro-Wilk normality test was used to test each parametric data set for normality. Since all data sets passed the normality test, a student's unpaired *t*-test (right-tailed, *α* = 0.05) was used to test for statistical significance of the difference between group means. In order to determine the clinical feasibility of using QUS as a cancer therapy monitoring system, multi-feature response classification was performed using a KNN classifier based on Euclidean distances (see Appendix). Rather than the absolute values, the changes in the QUS parameters relative to their pre-treatment value (week 0) were used as classification features, which are denoted here by the prefix Δ (i.e. ΔMBF). This baseline normalization was necessary to account for differences in the breast tissue echogenecity levels among the patients, owing to differences in breast densities. The imbalance in the data set was compensated for by randomly sampling (with replacement) from the responder group so as to have equal number of responders and non-responders (*N* = 16). Classification was performed 10 times (10 different responder group samples) with leave-one-patient-out evaluation. Due to the small number of features, an exhaustive search feature selection method was used for obtaining the optimal set of features for classification. The exhaustive search involved searching through all possible combinations of 2, 3, 4, 5, 6, and 7 parameters (120 combinations) in order to determine the minimal feature set resulting in the best classification accuracy, thereby removing any irrelevant or redundant parameters. Classification accuracy (number of correctly classified patient over the total number of patients) was used as the objective function to maximize. The metrics used for measuring classification performance were sensitivity, specificity, and accuracy.

### Quantitative ultrasound data analysis

All spectral analyses were carried out using the data from the −6 dB system transducer bandwidth, which was 3-8 MHz. The first step in the QUS analysis was computation of the attenuation coefficient estimate (ACE) of the tumor, which was used for attenuation correction of the tumor power spectrum. The ACE was computed using the reference phantom method (RPM) by estimating the rate of change in the spectral magnitude with depth and frequency relative to a reference medium with a known attenuation coefficient [33]. The reference material was an in-house constructed tissue-mimicking phantom with a measured attenuation coefficient of 0.15 dB/cm-MHz and a sound speed of 1515 m/s. The phantom was constructed based on [35], containing randomly dispersed glass microspheres with diameter of 18 (SD =3) μm and concentration of 2.2 g/L, in a 2% agar medium. Any phantom can be used for the RPM and for the QUS analysis performed here as long as it meets the following requirements. The phantom must produce a homogeneous speckle image and its BSC, speed of sound, and attenuation coefficient must be known (accurately measured). In order for system-dependent factors to be accurately corrected for using the RPM, the speed of sound of the reference should be matched to that of the sample [36]. Based on reported values [37], the speed of sound averaged over the fatty tissue, parenchyma, benign, and malignant lesion of the breast is 1490 m/s. Thus, the speed of sound of the phantom used here is within 1.5% difference of this value and is considered reasonable for use of the RPM.

In order to estimate ACE of a tumor ROI using the RPM, plots of phantom-normalized power spectrum amplitude versus depth were obtained by averaging the power spectra across laterally adjacent blocks and then plotting the average amplitude at each frequency against the depth of the blocks in the ROI. The mean ACE of the tumor was estimated by averaging the slopes of the linear fits to the amplitude versus depth data at all frequency points in the bandwidth. The newly found ACE was used to correct the tumor power spectrum for attenuation using the point attenuation compensation method [38]. The phantom power spectrum was corrected for using point attenuation compensation method and using the known attenuation coefficient (0.15 dB/cm-MHz). Afterwards, spectral parameters, including MBF, SI, and SS were determined from linear regression of the attenuation-corrected power spectrum within the usable (−6 dB) bandwidth. SI and SS are the intercept and slope parameters of the line of best fit, and MBF is the magnitude of the spectral fit at the center of the frequency bandwidth.

Using the same attenuation-corrected power spectrum, the backscatter coefficient (BSC) of the tumor was estimated using the reference phantom technique [39]. Then, by least-squares fitting of the Gaussian form factor model to the BSC, arguments of the Gaussian form factor, ASD and AAC, corresponding to the maximum coefficient of determination, R^2^, were determined. Details about scatterer size estimation can be found elsewhere [40].

Whereas spectral linear regression and BSC models are based on incoherent scattering, mean scatterer spacing (or spacing among scatterers) analysis techniques permit coherent scattering properties of tissues to be derived and coherent structures to be identified [41]. For SAS estimation, the power spectrum of the tumor was estimated using the autoregressive (AR) model and the AR model parameters were estimated using Burg's recursive algorithm [42]. For SAS computation, we estimated the power spectrum using the AR-method rather than the FFT since the former offers two advantages – it produces more conspicuous peaks, resulting in more accurate estimates of SAS, and it is less prone to ringing artifacts at small gate lengths [43]. The order of the AR model, p, was determined experimentally using an ultrasound image of an LABC patient's breast. The value was chosen by plotting the spectral autocorrelations (SAC) for a range of p values (from 10 to 100) and finding the p value at which the peak in the SAC was most conspicuous and did not contain multiple peaks. This value was experimentally determined to be 50 for the breast ultrasound data that was used in this study. The power spectrum was then normalized to that of a planar reflector. The planar reflector normalization at different depths was performed using pre-recorded reference RF data acquired from Plexiglas-water interface at six different depths (1-6 cm). For each RF block in the sample image, a reference RF block was selected by nearest neighbor approach. By computing the autocorrelation of the normalized power spectrum, the SAS parameter was determined from the frequency at which the peak occurred in the autocorrelation. The method used here for SAS estimation is described in detail in Tadayyon et al. [10].

### Classification system

After computing all 7 QUS features for all patients, classification was performed using the KNN classifier and using all possible combinations of QUS features. The KNN classifier determines the class of a point in the feature space based on the class which forms the majority of the points neighboring the point of interest and based on the distance between those points and the point of interest [44].
