# Correction: Quantitative ultrasound assessment of breast tumor response to chemotherapy using a multi-parameter approach

**DOI:** 10.18632/oncotarget.18068

**Published:** 2017-05-22

**Authors:** Hadi Tadayyon, Lakshmanan Sannachi, Mehrdad Gangeh, Ali Sadeghi-Naini, William Tran, Maureen E. Trudeau, Kathleen Pritchard, Sonal Ghandi, Sunil Verma, Gregory J. Czarnota

**Present:** The funding acknowledgements were omitted from the original paper.

**Correct:** The proper funding acknowledgements are given below.

Original article: Oncotarget. 2016; 7:45094-45111. doi: 10.18632/oncotarget.8862

